# Effects of mixing of europium oxide in resin composites on the fluorescence characteristics and mechanical properties

**DOI:** 10.1038/s41405-025-00304-0

**Published:** 2025-02-03

**Authors:** Yuta Utsumi, Masatsugu Oishi, Kazuhide Yonekura, Masaomi Ikeda, Yusuke Matsuki, Kenichiro Ohge, Tomoki Iuchi, Keiichi Hosaka

**Affiliations:** 1 Regenerative Dental Medicine, Tokushima University Graduate School of Biomedical Sciences, Tokushima, Japan; 2 Industrial and Social Science, Tokushima University Graduate School of Technology, Tokushima, Japan; 3 Department of Oral Prosthetic Engineering, Tokyo Medical and Dental University Graduate School of Medical and Dental Sciences, Bunkyo-ku, Tokyo Japan

**Keywords:** Composite resin, Bonded restorations

## Abstract

**Objective:**

The objective of this study is to disperse europium oxide (Eu_2_O_3)_ in a resin composite (RC) using a planetary centrifugal mixer and assess its effects on photoluminescence and mechanical properties.

**Materials and methods:**

A commercially available RC was mixed with Eu_2_O_3_ at various concentrations using a planetary centrifugal mixer. The fabricated samples were evaluated using scanning electron microscopy and a spectrofluorometer to assess their crystal structures, particle sizes, and photoluminescence properties. Vickers microhardness measurements were performed, along with a three-point bending test. Statistical analyses were performed to assess the mechanical properties.

**Results:**

The intensity of red fluorescence increased with the increase of europium oxide concentration. The fluorescence spectra at 613 and 620 nm exhibited higher intensities under excitation at 254 nm. Eu_2_O_3_ was dispersed in RC regardless of the Eu_2_O_3_ concentration, and no aggregation was observed. Regarding the mechanical properties, there were no significant differences in the flexural strength or modulus, and the Vickers hardness gradually increased with increasing Eu_2_O_3_ concentration.

**Conclusion:**

To mix Eu_2_O_3_ with RC, visible fluorescence emission was observed even with increasing the Eu_2_O_3_ concentration, and the mechanical properties of RC were unaffected. Based on our study, a 15 wt% concentration of Eu₂O₃ is the appropriate concentration, as it achieves strong fluorescence emission without compromising the mechanical properties or color tone of the RC.

## Introduction

Resin composite (RC) restorations, which minimize the amount of tooth structure removal, are widely recognized as clinically effective treatments because of their ability to minimize treatment time and risks [1]. Furthermore, the recently introduced universal-shade RC has enhanced color-matching capabilities, enabling aesthetic treatments in dentistry [2, 3]. While RC exhibits a white color and translucency close to that of natural teeth [4–6], challenges arise during its removal, making it difficult to distinguish with a tooth [7, 8]. Consequently, issues such as inadvertent tooth loss and reduced longevity due to residual material or over-reduction are of concern in dental treatments including endodontic and orthodontic treatments [9–12]. Therefore, it is necessary to develop alternative noninvasive methods that can visually detect the RC. Current examination methods utilize X-ray irradiation, which can be harmful [13]. An alternative method involves optical coherence tomography (OCT), employing near-infrared light. In contrast to X-rays, OCT is safe for vulnerable groups, such as infants and pregnant women; however, the depth detection limit is typically several millimeters [14].

A method utilizing ultraviolet (UV) irradiation has been proposed for visual detection that exploits the differences in light transmittance and fluorescence generation between the fillers in RC and the tooth [15]. Therefore, we focused on the fluorescent filler europium oxide (Eu_2_O_3_), which is used as a dental aesthetic filler to adjust the fluorescence of RC. Eu_2_O_3_ has been explored as an aesthetic filler in dental resin composites to enhance optical properties, which plays a key role in mimicking the natural appearance of teeth. Eu_2_O_3_ emits red fluorescence at 613 nm upon excitation at 254 nm, improving restoration visualization, maintaining aesthetic properties, and enhancing radiopacity for clinical evaluation [16, 17]. These advantages make Eu_2_O_3_ a promising material, as it offers benefits beyond the mechanical improvements provided by conventional fillers (e.g., silica, zirconia). However, studies have indicated that Eu_2_O_3_ exhibits weak fluorescence in commercial composites and may potentially affect polymerization efficiency, raising concerns about its impact on material performance [7, 18]. Additionally, studies have confirmed that increasing the Eu_2_O_3_ concentration would increase the fluorescence intensity; however, increasing the Eu_2_O_3_ concentration becomes aggregated and causes the degradation of the mechanical properties [18]. Eu_2_O_3_ has been mixed into RC by hand, which is impractical for commercial usage. In addition, the states of Eu_2_O_3_ in RC are not uniform. Therefore, considering industrial applications, a planetary centrifugal mixer has also been used to mix Eu_2_O_3_ in RC, which can facilitate dispersion and reduce particle aggregation [19, 20]. Compared to other mixing instruments, the planetary centrifugal mixer stands out for its ability to achieve uniform particle dispersion, minimize air bubble formation, and prevent contamination through sealed, non-contact mixing. Its effectiveness with highly viscous materials, such as RCs, makes it particularly suitable for industrial applications where these factors are crucial. However, the effects of using a planetary centrifugal mixer to mix Eu_2_O_3_ with RCs on the photoluminescence and mechanical properties of the mixture have not been clarified. Therefore, this study investigates the effects of mixing Eu_2_O_3_ in RCs on the fluorescence characteristics and mechanical properties.

## Materials and methods

### Materials

The RC (Clearfil Majesty ES Flow Universal High; Kuraray Noritake Dental Inc., Tokyo, Japan) and red-light-emitting Eu_2_O_3_ (99.9% Europium oxide; Kojundo Chemical Laboratory, Saitama, Japan) were mixed using a planetary centrifugal mixer (ARE-310; Thinky Corporation, Tokyo, Japan). This system could mix the samples uniformly and efficiently with a consistent deformation process [19]. Eu_2_O_3_ was mixed with RC at concentrations of 5, 10, 15, 20, and 25 wt%, and an RC sample without Eu_2_O_3_ was also prepared.

### Visual evaluation

The Eu_2_O_3_-mixed RC samples at each concentration were filled into disc-shaped molds (φ16 mm; thickness = 2.0 mm). The mixtures in these molds were light-cured using a light-emitting diode (LED) curing unit (PenCure 2000; Morita, Osaka, Japan) with a radiant intensity of 2000 mW/cm. The RC parts were irradiated at the center, moving 5 s each to the top, bottom, left, and right to overlap with previously irradiated RC parts. The RC samples were removed from the molds, inverted, and again light-cured for 25 s. The fluorescence of the RC samples retrieved from the molds was observed in a dark box under 254 and 365 nm UV lamps [21]. Additionally, a colorimeter (VITA Easyshade V; Hakusui Trading Corporation, Osaka, Japan) capable of quantifying subtle color differences, ΔEab, was used to measure the color differences under a fluorescent lamp with a black background [22]. The differences in fluorescence with UV light and color variations under a fluorescent bulb were assessed for the RC samples at each concentration.

### Crystal structure, particle size, and photoluminescence properties

The crystal structure, particle size, and photoluminescence properties of the Eu_2_O_3_-mixed RC samples were evaluated using X-ray diffraction (XRD), scanning electron microscopy (SEM), and spectrofluorometry, respectively. The XRD measurements were conducted using an X-ray diffractometer (Mini Flex 600; Rigaku Corporation, Tokyo, Japan) [23]. The particle morphology of Eu_2_O_3_ and its dispersion in RC samples were examined using SEM (SU3500; Hitachi High-Tech Corporation, Tokyo, Japan). The particle diameters of Eu_2_O_3_ were measured using ImageJ software (National Institutes of Health, Bethesda, Maryland, USA) with SEM images [24]. The photoluminescence spectra of the Eu_2_O_3_-mixed RC samples were measured at 23 °C using a spectrofluorometer (FP-8550; Jasco Corporation, Tokyo, Japan) equipped with a Xe lamp as the excitation source [25].

### Mechanical properties

The flexural strength (Fs) and modulus (Fm) were measured via a three-point bending test using a tabletop testing machine (EZ-SX; Shimadzu Corporation, Kyoto, Japan). Initially, the RC samples were filled into beam-shaped silicon molds (1.0 × 1.0 × 10.0 mm^3^) [26]. The RC samples in the mold were exposed to an LED curing unit (tip diameter: 15.0 mm). The RC samples were irradiated by moving them from the center to the left and right for 5 s each, ensuring an overlap with the pre-irradiated part, which resulted in a total light-curing time of 25 s. The samples were then removed from the molds, inverted, and again light-cured for 25 s. After immersion in pure water at 37 °C for 24 h, all the beam-shaped RC samples underwent the three-point bending test, where Fs and Fm were measured using a tabletop testing machine at a displacement speed of 1 mm/min.

Fm was calculated as follows:

1 $${{{\rm{Fm}}}}={L}^{3}\varDelta P/4b{h}^{3}\varDelta d$$

where *L* represents the span between the support rods (5 mm), *h* represents the height of the RC samples, *b* represents the width of the RC samples, and *ΔP* and *Δd* represent the incremental changes in load and deflection, respectively, between specific points within the elastic region of the curve.

Fs was determined as follows:

2 $${{{\rm{Fs}}}}=3FL/2b{h}^{2}$$

where *F* represents the load at the point of failure.

The samples used for Vickers microhardness measurements were prepared similarly to those for visual evaluation. After immersion in pure water at 37 °C for 24 h, they were polished with 240, 400, and 800 grit sandpaper. The Vickers microhardness tests were performed using a Microhardness Tester (MVK-E; Akashi Seisakusho Ltd., Tokyo, Japan) with a load of 2.942 N applied for 10 s [27].

The Vickers hardness (Hv) was calculated as follows:

3 $${{{\rm{Hv}}}}=0.1891\,\times \,F/{d}^{2}$$

where *F* represents the load (N) and *d* represents the diameter (mm).

### Statistical analysis

The determined sample size for each group was in accordance with the standardized protocol of ISO4049:2019. According to ISO4049;2019, the minimum required sample size for mechanical properties is 5. In this experiment, the sample size in Fs and Fm was determined using the sample size calculator software program (G. power 3.1.9.7). The statistical power (1- b) at 0.80, the effect size at 0.5, and the significance level (a) was set at 0.05. Therefore, the minimum sample size estimated for this study was 10 samples in each group.

Next, Sample size in Hv was calculated using the sample size determination method for two-tailed *t*-tests as follows:

$$n=2\times {(1.96+0.84)}^{2}\times {({Standard}\, {Deviation})}^{2}/(Mean\,Difference)$$

Standard deviation (SD ≈ 1.78) and the difference between the mean (Δ ≈ 2.79) of each concentration groups were obtained from the data of the study, then the number of specimens per group was set to 6.

Fs was evaluated using a *t*-test (Welch’s method) with the Bonferroni correction, and Fm was assessed using the Kruskal–Wallis test. The Vickers hardness was evaluated using a Dunn’s test with the Bonferroni correction.

## Results

### Visual evaluation

**Fig. 1:**
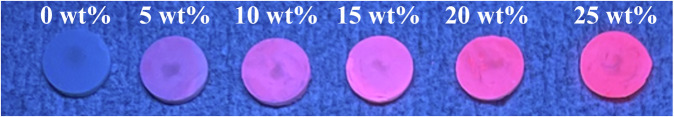
Photographs under UV-C (254 nm) illumination of the RC samples. UV ultraviolet, RC resin composite.

**Fig. 2:**
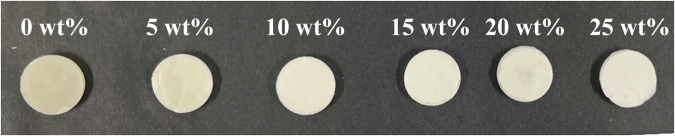
Photographs under fluorescent-bulb illumination of the RC samples. RC resin composite.

Figure 1 shows an image of the Eu_2_O_3_-mixed RC samples under 254 nm irradiation. The red color is stronger for the RC samples with higher Eu_2_O_3_ concentrations. The RC sample without Eu_2_O_3_ reflected the UV light, whereas the Eu_2_O_3_-mixed RC samples with Eu_2_O_3_ concentrations of 5–10 wt% showed a purplish color that changed to a reddish color as the Eu_2_O_3_ concentration increased. A pronounced red color was observed for the RC samples with Eu_2_O_3_ concentrations of ≥15 wt%. However, under 365 nm irradiation, increasing the concentration of Eu_2_O_3_ did not result in the photoluminescence properties observed under 254 nm. Observations of the RC samples under a fluorescent-bulb lamp (approximately 4000–5000 K) revealed a whitening of the color tone with an increase in the Eu_2_O_3_ concentration (Fig. 2). Color differences under the fluorescent-bulb evaluated using a colorimeter showed no significant difference in color up to a mixing concentration of 15 wt%. However, significant color differences relative to 0 wt% were observed above 20 wt% (Table 1).

**Table 1:** Color differences of the RC samples under fluorescent-bulb illumination at each concentration.

	5 wt%	10 wt%	15 wt%	20 wt%	25 wt%
ΔEab	12.3	13.3	13.6	19.4	25.0

ΔEab shows the different colors between RC (0 wt%) and each RC sample.

*RC* resin composite.

### Crystal structure, particle size, and photoluminescence properties

**Fig. 3:**
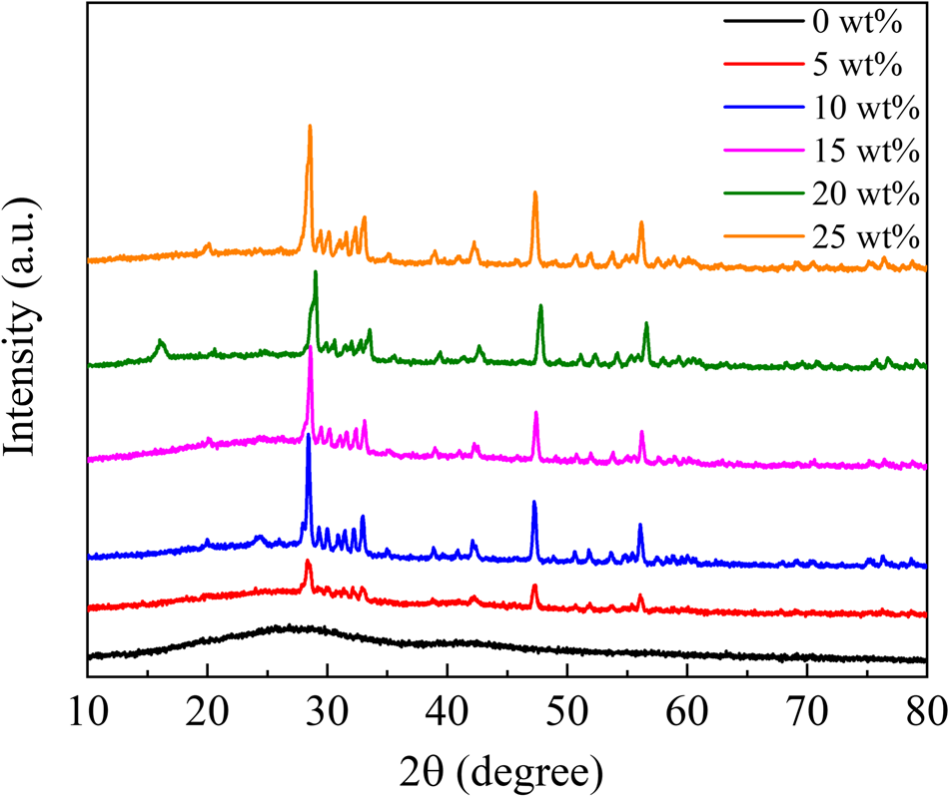
XRD profiles of the RC samples. XRD X-ray diffraction, a.u. arbitrary unit, RC resin composite.

**Fig. 4:**
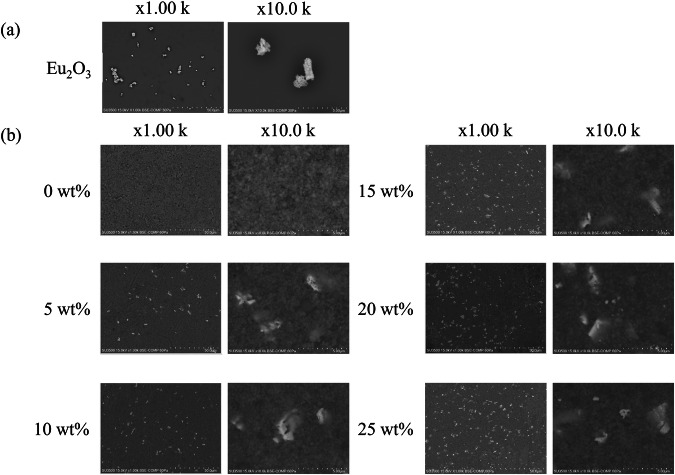
SEM images. **a** SEM images of Eu_2_O_3_ and **b** the RC samples. Eu_2_O_3_ europium oxide, SEM scanning electron microscopy, RC resin composite.

**Fig. 5:**
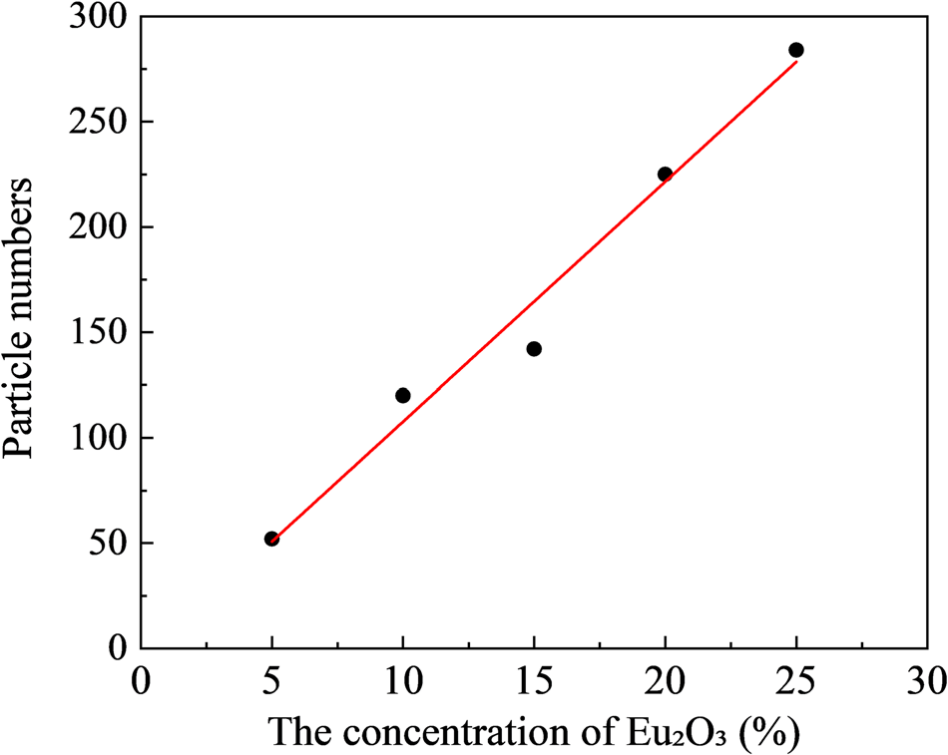
Number of fluorescent filler particles in the RC samples. Eu_2_O_3_ europium oxide, RC resin composite.

**Fig. 6:**
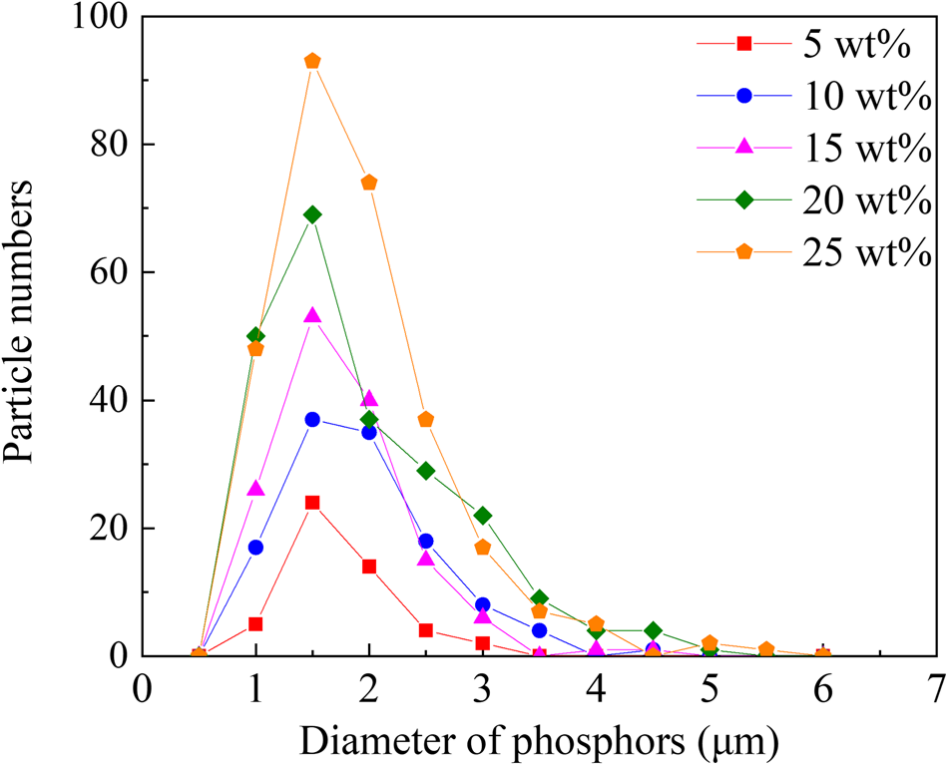
Particle size distribution of fluorescent filler in the RC samples. RC resin composite.

Figure 3 shows the XRD profiles of the RC samples. The XRD profiles of the Eu_2_O_3_-mixed RC samples and Eu_2_O_3_ powder matched, indicating that the crystal structure of Eu_2_O_3_ remained unchanged upon mixing with the RC. Figure 4a presents SEM images of Eu_2_O_3_, showing particles of several micrometers. Figure 4b presents SEM images of the RC samples showing submicron-sized particles distributed in the RC samples, increasing in quantity with increasing the Eu_2_O_3_ concentration. The distribution of particles in RC remained consistent regardless of the Eu_2_O_3_ concentration, and no particle aggregation was observed. Figure 5 shows that the particles distributed within the material exhibit an increase with increasing the Eu_2_O_3_ concentration. Figure 6 shows that the particle size distribution does not change significantly with increasing the Eu_2_O_3_ concentration.

**Fig. 7:**
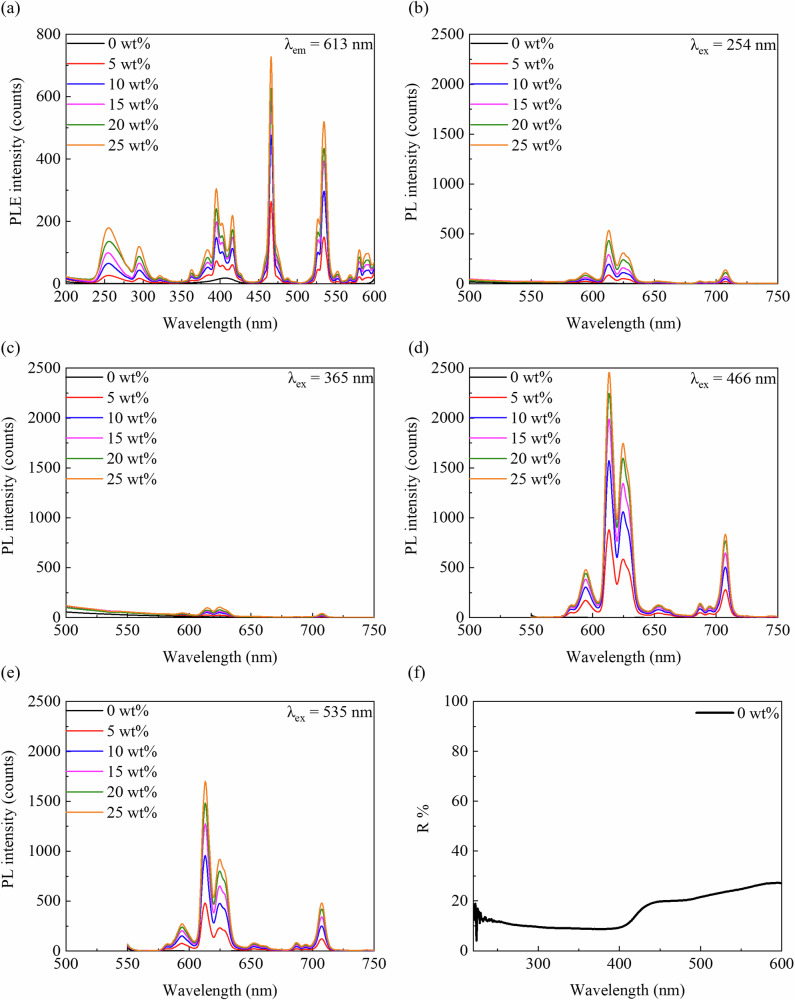
Excitation spectrum, Emission spectra, and Reflectance spectrum. **a** Excitation spectrum at 613 nm emission of the RC samples. Emission spectra at **b** 254, **c** 365, **d** 466, and **e** 535 nm excitation. **f** Reflectance spectrum of RC (0 wt%). PL photoluminescence, PLE photoluminescence excitation, R reflection, RC resin composite.

Figure 7 shows the excitation and fluorescence spectra. In Fig. 7a, the excitation spectrum at 613 nm revealed a strong peak at 254 nm, corresponding to visible red fluorescence, whereas the weaker peak at 365 nm does not correspond to red fluorescence. Figure 7b–e shows the emission spectra under different excitation wavelengths, where the peak intensities increase with the Eu_2_O_3_. Figure 7b, c shows stronger fluorescence at 613 and 620 nm under excitation at 254 nm than at 365 nm. Additionally, longer excitation wavelengths (466 and 535 nm) yielded similar emission spectra, with peak intensities higher than those at shorter wavelengths. Figure 7f shows the reflectance of the RC without Eu_2_O_3_, indicating a lower reflectance at 200–400 nm and a higher reflectance beyond 400 nm.

### Mechanical properties

Table 2 presents the Fs and Fm values of the Eu_2_O_3_-mixed RC samples. No significant differences were observed even with an increase in concentration to 25 wt%, and no statistical significance was found. The Vickers hardness was also evaluated for the RC samples. The addition of Eu_2_O_3_ gradually increased the Vickers hardness of the RC samples. In particular, a significant increase was observed with the addition of ≥15 wt% Eu_2_O_3_ in comparison with 0 wt% (Table 3). Additionally, when the mold was filled with the RC samples at concentrations of ≥30 wt%, sufficient curing did not occur even at extended light-curing times exceeding the required time, preventing the fabrication of the RC samples. Therefore, the threshold for light-curing is suggested to be 25 wt%.

**Table 2:** Flexural strength and modulus of the RC samples.

	Fs [MPa]	Fm [MPa]
0 wt%	143.8 (14.7)^*^	2772 (330)^**^
5 wt%	137.4 (7.9)^*^	2919 (403)^**^
10 wt%	151.9 (17.4)^*^	2826 (314)^**^
15 wt%	135.9 (11.3)^*^	2883 (245)^**^
20 wt%	140.3 (11.5)^*^	2922 (260)^**^
25 wt%	140.2 (13.3)^*^	3186 (280)^**^

Values are expressed as mean (S.D.) (*n* = 10), and identical superscripts indicate no statistically significant differences (*P* > 0.05). Fs was evaluated using a *t*-test (Welch’s method) with the Bonferroni correction, and Fm was assessed using the Kruskal–Wallis test.

*Fs* Flexural Strength, *Fm* Flexural Modulus, *RC* resin composite.

**Table 3:** Vickers hardness of the RC samples.

	Hv
0 wt%	34.27 (0.88)^a^
5 wt%	37.06 (1.78)^abc^
10 wt%	37.35 (0.80)^abc^
15 wt%	39.53 (1.12)^bcd^
20 wt%	40.34 (1.07)^cd^
25 wt%	41.13 (0.38)^d^

Values are expressed as mean (S.D.) (*n* = 6), and different superscripts indicate statistically significant differences between groups (*P* < 0.05). The Vickers hardness was evaluated using a Dunn’s test with the Bonferroni correction.

*Hv* Vickers hardness, *RC* resin composite.

## Discussion

In terms of visual evaluation, visual inspection under UV-C illumination, as shown in Fig. 1, revealed a strong red color with an increase in the Eu_2_O_3_ concentration. The observation of visible red fluorescence was challenging at low concentrations (0–10 wt%); however, distinguishable red luminescence was observed beyond 15 wt%. Furthermore, as the Eu_2_O_3_ concentration increased, the RC samples, as shown in Fig. 2, became whiter. Sufficient light-curing did not occur when Eu_2_O_3_ was mixed at concentrations exceeding 30 wt%. As shown in Fig. 7a–e, the increase in fluorescence intensity was attributed to the dependence of fluorescence on the Eu_2_O_3_ concentration. Factors contributing to the observed whitening effect in the RC samples under visual inspection included the increased density of the Eu_2_O_3_ particles (the density of e Eu_2_O_3_ is 7.42 g/cm³) and the increase in refractive-index differences. Translucency and color tone are affected by factors such as the type and size of the matrix resin and filler, density, and refractive indices [28]. Additionally, an increase in the Eu_2_O_3_ concentration resulted in the partial reflection of incident light on the RC surface as well as light scattering, as shown in Fig. 7f, which reduced the intensity and transparency of the incident light. This may suppress the light-curing of the RC samples.

Next, we consider the crystal structure of the investigated systems. From the XRD profiles shown in Fig. 3, the Eu_2_O_3_ powder used in this study was a mixture of cubic and monoclinic phases. The strong XRD peaks correspond to the cubic phase, and some minor peaks correspond to the monoclinic phase. The 0 wt% sample shows no significant peaks, suggesting the absence of crystalline Eu_2_O_3_ powders in the control sample. The XRD profiles (Fig. 3) stayed unchanged and no additional peak was observed upon the addition of Eu_2_O_3_, which indicated that Eu_2_O_3_ is compatible with RC. Because the crystal structure of the Eu_2_O_3_ particles in the RC samples remained unchanged, the Eu_2_O_3_ particles exhibited stability toward the frictional heat and pressure generated by the planetary centrifugal mixer. Furthermore, considering the particle size, as shown in Fig. 6, the absence of changes in the particle size distribution with an increasing Eu_2_O_3_ concentration indicates that the Eu_2_O_3_ particles were dispersed without aggregation. Furthermore, even at increased concentrations, the majority of the particles were distributed at a size of 1.5 μm.

As shown in Fig. 7b, c, comparing the observations of red fluorescence at excitation wavelengths of 254 and 365 nm revealed that the emission peak intensity was lower at 365 nm. As the Eu_2_O_3_ concentration increased, as shown in Fig. 7a, both the excitation and fluorescence spectra were enhanced, suggesting that the Eu_2_O_3_ concentration may be adjusted for visualization. As shown in Fig. 7d, e, strong emission peaks were observed at excitation wavelengths of 466 and 535 nm; however, these visible-range wavelengths may pose challenges for visual observations owing to potential interference with the light-curing units. Therefore, the optimal excitation wavelength for visual observations without considering the effects of the external environment, such as the treatment conditions or equipment, was 254 nm.

Regarding the mechanical properties, as shown in Table 2, no significant differences were observed in Fs or Fm as the Eu_2_O_3_ concentration increased up to 25 wt% in the RC samples. Additionally, the primary filler particle size (approximately 1.5 μm) fell within the range of nanocluster characteristics [29], and it did not affect Fs or Fm, which characterize the elasticity of the resin material. Furthermore, the reduction in the surface hardness suggests excessive polymerization shrinkage [27]. A higher degree of conversion generally correlates with a denser, more cross-linked polymer network, which directly impacts mechanical properties, such as Vickers hardness. Therefore, the inclusion of Eu_2_O_3_ in the RC increased the degree of hardening, ensuring complete polymerization. As shown in Table 3, the Vickers hardness values increased with higher Eu_2_O_3_ concentrations, suggesting that the presence of Eu_2_O_3_ may have positively influenced the polymerization process. The use of a planetary centrifugal mixer for mixing revealed no significant differences in Fs or Fm, and the microhardness increased.

This study has several limitations. First, the absence of UV-C illumination devices for clinical visualization represents the limitation of this study. Therefore, the challenge of developing UV-C illumination devices remains. Second, the study does not investigate the effects of various mixing methods or experimental polymer blends. Third, advanced instrumentation for optical and colorimetric properties was not utilized. Future research is recommended to address these limitations by focusing on the development of UV-C illumination devices, exploring various mixing methods, conducting polymer blend experiments, and employing advanced measurement techniques for optical and colorimetric properties.

## Conclusion

This study suggests that mixing Eu_2_O_3_ with RC results in observable fluorescence emission, even with increased Eu_2_O_3_ concentrations, while the mechanical properties of RC remain unaffected. Based on our study, a 15 wt% concentration of Eu_2_O_3_ is the appropriate concentration, as it achieves strong fluorescence emission without compromising the mechanical properties or color tone of the RC. Additionally, the Eu_2_O_3_ concentration can be adjusted to meet specific application requirements, providing flexibility for customization. In conclusion, the Eu_2_O_3_-mixed RC provides a promising solution for future practical applications.

## Data Availability

The datasets used and/or analyzed during the current study are available from the corresponding author upon reasonable request.
